# Photodynamic Activation of Cholecystokinin 1 Receptor Is Conserved in Mammalian and Avian Pancreatic Acini

**DOI:** 10.3390/biomedicines11030885

**Published:** 2023-03-13

**Authors:** Jie Wang, Zong Jie Cui

**Affiliations:** Institute of Cell Biology, Beijing Normal University, Beijing 100875, China; 15321227265@126.com

**Keywords:** photodynamic action, cholecystokinin 1 receptor, pancreatic acini, rat, mouse, Peking duck

## Abstract

Cholecystokinin 1 receptor (CCK1R) is the only G protein coupled receptor that is activated in type II photodynamic action, but whether this is a property common to both mammalian and avian species is not known. In this work, pancreatic acini were isolated from the rat, mouse, and Peking duck, and photodynamic CCK1R activation was examined. Isolated pancreatic acini were exposed to photosensitizer sulphonated aluminum phthalocyanine (SALPC) and photodynamic action elicited by a brief light-emitting diode (LED 675 nm) pulse (1.5 min); photodynamic CCK1R activation was assessed by Fura-2 fluorescent calcium imaging. Photodynamic action was found to induce persistent calcium oscillations in rat, mouse, and Peking duck pancreatic acini, with the sensitivity order of mouse > rat > Peking duck. Photodynamically-activated CCK1R could be inhibited reversibly by CCK1R antagonist devazepide (1 μM); photodynamic CCK1R activation was blocked by pre-incubation with ^1^O_2_ quencher Trolox C (300 µM). The sensitivity of photodynamic CCK1R activation was correlated with the increasing size of the disordered region in intracellular loop 3. These data suggest that photodynamic CCK1R activation is conserved in both mammalian and avian species, as evidenced by the presence of the photodynamic activation motif “YFM” in transmembrane domain 3.

## 1. Introduction

The minimal functional unit of all organisms is the individual cell, which executes specific functions via functional proteins such as G protein coupled receptors (GPCR), ion channels, transporters, enzymes, and others [1]. Conventional receptor pharmacology is based on non-covalent, ligand-specific modulation of resting receptor activity, including both orthosteric and allosteric ligand regulations [2,3,4]. In recent years, ligand-independent receptor regulation, especially of GPCR, has emerged prominently, such as quenching of receptor activation by neutrophil respiratory burst [5], by neutrophil extracellular trap components (such as extracellular histones) [6], by hydrophobic bile acids [7], and by cholesterol density in the plasma membrane [8,9]. Most remarkably, the cholecystokinin 1 receptor (CCK1R), a rhodopsin-like or class A GPCR family member, is permanently activated in photodynamic action [10,11,12,13,14,15].

CCK1R is unique among GPCR of all classes in that it is activated permanently in a type II photodynamic action (photon-to-photosensitizer energy-transfer process involving photon-absorbing macromolecular photosensitizer, light of wavelength at maximal absorption by photosensitizer, and ground state oxygen) by the excited delta singlet oxygen (Δ^1^O_2_) [13,14,15]. The initial hint for this unique pharmacological property of CCK1R might have been obtained from photo-affinity labeling experiments performed by the Jamieson laboratory at Yale [16]. The photoaffinity probe 2-nitro-5-azidobenzoyl-glycine-CCK-8 (NAB-Gly-CCK-8) was found, after multiple cycles of ultraviolet A (UVA) photolysis (>320 nm), to irreversibly elicit amylase secretion in the isolated guinea pig pancreatic acini [16]. It has been subsequently reported that photodynamic action, with either Rose Bengal [17] or with sulphonated aluminum phthalocyanine (SALPC) [18] as the photosensitizer, stimulated amylase secretion from freshly isolated rat pancreatic acini, when the pancreatic acinar cell plasma membrane remained completely intact [17,18]. In perifused isolated rat pancreatic acini, photodynamic action with SALPC or gadolinium porphyrin-like macrocycle B (PLMGdB) as a photosensitizer was found to elicit persistent cytosolic calcium oscillations, which highly resembled stimulation with picomolar CCK (namely, physiological concentrations), with the exception that the calcium oscillations could not be washed out after photodynamic action, i.e., calcium oscillations persisted [10,11]. Photodynamically induced persistent calcium oscillations were blocked specifically by CCK1R antagonist FK480 in isolated rat pancreatic acini, confirming that rat CCK1R was activated in photodynamic action with SALPC as the photosensitizer [12].

Other than in normal, physiologically functioning pancreatic acini, CCK1R in rat pancreatic acinar tumor cell line AR4-2J was also found to be photodynamically activated, with genetically encoded protein photosensitizer KillerRed [13]. In fact, all genetically encoded protein photosensitizers (GEPP) reported in the literature were found to photodynamically activate CCK1R in AR4-2J cells [19], with either an external light source or with bioluminescence emitted from simultaneously expressed NanoLuc to drive photodynamic action [19,20].

Interestingly, it was found that the human CCK1R, when heterologously expressed in HEK-293 cells (i.e., outside of the microenvironment of pancreatic acinar cell plasma membrane), was activated permanently in photodynamic action, with SALPC as the photosensitizer [13]. Protein photosensitizer KillerRed or miniSOG tagged either to the N- or C-terminus of the human CCK1R directly, or with a (GSG)_n_ linker of determined length (n = 4, 8) in between the protein photosensitizer miniSOG and N-terminus of CCK1R, was able to photodynamically activate in-frame human CCK1R [14]. Photodynamic CCK1R activation was found to be critically dependent upon the third transmembrane domain (TM3) of human CCK1R. Most remarkably, TM3 of CCK1R, when transplanted to the muscarinic acetylcholine 3 receptor (M3R), could confer upon M3R the property of photodynamic activation, which was absent in the native M3R [12,15]. Photodynamic CCK1R activation was found to be accompanied by a dimer-to-monomer transition of the CCK1R protein molecule, which was partially purified from isolated rat pancreatic acini [21], a monomerization process similar to CCK-stimulated rat CCK1R dimer-to-monomer transition, as monitored by bioluminescence resonance energy transfer (BRET) in between CCK1R protein molecules expressed in COS-1 cells [22].

It is now established that CCK1R could be readily activated in photodynamic action with varied photosensitizers including both chemical photosensitizers such as SALPC and genetically encoded protein photosensitizers such as KillerRed and miniSOG [13,19]. However, does this property of photodynamic activation of human and rat CCK1R extend to CCK1R in other species?

Therefore, in the present work, photodynamic CCK1R activation was compared in freshly isolated rat, mouse, and Peking duck pancreatic acini. It was found that CCK1R could also be photodynamically activated in mouse and Peking duck pancreatic acini, with the sensitivity order of mouse > rat > Peking duck. A detailed comparison of rat, mouse, and Peking duck CCK1R sequence and structure revealed a high level of similarities, including conservation of the photodynamic activation motif of “YFM”; further, the size of the disordered region in the third intracellular loop (ICL3) of CCK1R could be correlated to the sensitivity of their photodynamic activation.

## 2. Materials and Methods

### 2.1. Materials

Sulfated cholecystokinin octapeptide (CCK, #1166) and CCK1R antagonist devazepide (#2304) were from Tocris Cookson (Bristol, UK). Minimum essential medium (MEM) amino acid mixture (50×, #11130051) was from Thermo Scientific (Shanghai, China). Fura-2 AM (#21020) was from AAT Bioquest (Sunnyvale, CA, USA). Goat anti-human CCK1R polyclonal primary antibody (#ab77269) (against synthetic peptide of human CCK1R^242−257^) and tetramethylrhodamine isothiocyanate (TRITC)-conjugated donkey anti-goat secondary antibody (#ab6738) were from Abcam (Cambridge, UK). Cell-Tak (#354241) was from BD Bioscience (Bedford, MA, USA). Sulfonated aluminum phthalocyanine (SALPC, #AlPcS 834) was from Frontier Scientific (West Logan, UT, USA). (±)-6-Hydroxy-2,5,7,8-tetramethylchromane-2-carboxylic acid (Trolox C, #238813) and soybean trypsin inhibitor (#T9128) were from Sigma (St. Louis, MO, USA). Collagenase P (#11213873001) was from Roche (Mannheim, Germany). 4-(2-Hydroxyethyl) piperazine-1-ethanesulfonic acid and N-(2-Hydroxyethyl) piperazine-N′-(2-ethanesulfonic acid) (HEPES, #391338) were from Merck (Darmstadt, Germany). Restriction enzymes (EcoRI, #1611 and XhoI, # 1635) were from Takara (Beijing, China). HiPure Total RNA Plus Mini Kit (#R4121-02) was from Magen (Guangzhou, China). The 2×Taq Master Mix (#P112-01) was from Vazyme (Nanjing, China). GoScript Reverse Transcription Kit (#238813) was from Promega (Shanghai, China). GoldView Molecular Markers (GV-2) was from SaiBaiSheng (Beijing, China).

### 2.2. Isolation of Rat, Mouse, and Peking Duck Pancreatic Acini

Rat pancreatic acini were isolated from male Sprague-Dawley rats (250–450 g in body weight) by collagenase P digestion in Krebs buffer, as reported [10,12]. The pancreata were excised, infiltrated with collagenase P-containing buffer (0.2 g·L^−1^, 10 mL) before digestion in a shaking water bath (37 °C, 120 cycles per min) for 10 min, followed by an additional 20 min digestion in fresh collagenase P-containing buffer. The digested tissue was dispersed with a plastic pipette, filtered through a nylon mesh (150 mesh), layered onto buffer containing 3.8% BSA, then acini were allowed to gravitate to the bottom of the 10 mL test tube. The rat pancreatic acini pellet obtained were then re-suspended, centrifuged (20 g, 1 min), and washed three times.

Krebs buffer used for collagenase P digestion had the following composition (in mM): NaCl 118, KCl 4.7, CaCl_2_ 2.5, MgCl_2_ 1.13, NaH_2_PO_4_ 1.0, D-glucose 5.5, HEPES 10, L-glutamine 2.0, BSA 20 g·L^−1^, MEM amino acid mixture (50×) 2%, and soybean trypsin inhibitor 0.1 g·L^−1^. Buffer pH was adjusted to 7.4 with NaOH 4 M and oxygenated with O_2_ 100%. L-glutamine, BSA, amino acid mixture, and soybean trypsin inhibitor were omitted when buffer was used for perifusion and calcium imaging. Pancreatic acini perifusion was performed at a rate of 1 mL.min^−1^.

Mouse pancreatic acini were isolated similarly by collagenase P (0.2 g.L^−1^, 10 mL) digestion, from male Kunming white mice (body weight 20–35 g). The pancreas was excised and injected with collagenase P-containing buffer for digestion in a shaking water bath sequentially for 10 and 15 min. The digested pancreatic mince was dispersed, filtered (150 mesh), passed through buffer containing 3.8% BSA, centrifuged (20 g, 1 min), and washed three times.

Peking duck pancreatic acini were similarly isolated by collagenase P digestion from Peking duck (*Anas platyrhynchos domestica*) (body weight 250–450 g) [23]. The spleen lobe of the pancreas was excised, infiltrated with collagenase P-containing buffer (0.4 g·L^−1^, 10 mL), and sliced with a razor blade, before sequential collagenase P digestion (10, 15 min sequentially). Digested pancreatic tissue was dispersed, filtered (150 mesh), and passed through buffer containing 3.8% BSA. The Peking duck pancreatic acini obtained were centrifuged (35 g, 1 min) and washed three times.

The above isolated rat, mouse, and Peking duck pancreatic acini were incubated in Krebs buffer for 30 min for recovery before experimentation (CCK stimulation, photodynamic action, immunocytochemistry, or for total RNA extraction for CCK1R gene cloning from the Peking duck pancreatic acini).

Rats, mice, and Peking ducks used for pancreatic acini isolation were maintained under a natural light/dark cycle, with commercial food and tap water fed ad libitum, and killed by CO_2_ asphyxia. The experimental protocol was approved by Animal Use and Ethics Committee, College for Life Sciences, Beijing Normal University (CLS-EAW-2017-015).

### 2.3. The Cloning of CCK1R Gene from Peking Duck Pancreatic Acini

Total RNA was extracted from freshly isolated Peking duck pancreatic acini with HiPure Total RNA Plus Mini Kit as per the manufacturer’s instructions. RNA concentration was determined in a Nanodrop2000 spectrometer (Thermo Fisher Scientific, Wilmington, DE, USA). mRNA was reverse-transcribed (RT) with a GoScript Reverse Transcription Kit to obtain cDNA. To a polymerase chain reaction (PCR) tube, Oligo (dT) 1 µL and RNA 1 µg were added, denaturation was performed at 70 °C for 5 min. It was then cooled on ice for 5 min before the addition of the following: reaction buffer (×5) 5 µL, MgCl_2_ 2.5 µL, RNAase inhibitor 0.5 µL, M-MLV reverse transcriptase 1.25 µL, and dNTP (10 mM) 1.25 µL, topped up with diethylpyrocarbonate (DEPC)-treated water to 25 µL. Reverse transcription was performed at two temperatures sequentially: 40 °C for 60 min and then 70 °C for 15 min to obtain cDNA. PCR reactions were then performed in a solution composed of 2 µL cDNA template, 15 µL 2×Taq Master Mix, and primers (forward and reverse) 1 µL, topped up with diethylpyrocarbonate (DEPC)-treated water to 30 µL. PCR was carried out: initial denaturation at 95 °C for 5 min, followed by 35 cycles at 95 °C for 30 s, 60 °C for 30 s, and 72 °C for 1.5 min, followed by one cycle at 72 °C for 5 min for final prolongation. The PCR primers based on the CCK1R sequence of mallard duck (*Anas platyrhynchos*) were as follows: forward primer 5′-ATGGTTAAAGAGCTTACTTC-3′, reverse 5′-TCAGGGGGGTGCAGAGATATGT-3′. RT-PCR products were run on 1% agarose gel with 0.01% GoldView added, 120 V, 40 min before imaging.

After PCR product verification by agarose gel electrophoresis, target bands in the agarose gel were cut off and collected, and PCR product was recovered with TIANgel Midi Purification Kit (TIANGEN, #DP209-02) as described in the users’ instructions manual.

The recovered PCR DNA product was ligated to T vector (pMD19): DNA product 5 µL, T vector 1 µL, and solution I 5 µL were mixed and placed in a metal bath (16 °C) overnight. Positive ligation products were detected on agarose gel.

The ligation products were transformed into *E. coli* (Top10 competent cells). TOP10 cells (50 µL) from refrigerator (−80 °C) were thawed on ice, incubated sequentially on ice with ligation products (30 min), in a water bath (42 °C) for 45 s, on ice for 3 min. Sterile LB medium (450 µL) was added and shake incubated (37 °C) for 50 min. The bacteria suspension (100 µL) was evenly placed onto LB plates (Φ9 cm) containing Amp^+^ and incubated (>12 h) before resistant strains appeared. Colonies were picked and cultured in liquid LB ^+^ Amp^+^ medium (>12 h). The resistant *E. coli* suspension was used for PCR verification. PCR experiments were performed as described above, but with annealing at 59 ℃. Boiled bacteria (200 µL) were used as a DNA template. Universal primers of M13-47 (TGTAAAACGAC-GGCCAGT) and RV-M (CAGGAAACAGCTATGACC) were used as forward and reverse primers, respectively. PCR products were run on agarose gel, and bacteria suspension showing target DNA stripe was sent for DNA sequencing (RuiBiotech, Beijing, China).

DNAman 8.0 was used to concatenate the forward and reverse sequences to obtain the complete sequence. An ORF finder was used for gene sequence discovery, and TMHMM was used for putative protein transmembrane domain prediction.

The predicted Peking duck (*Anas platyrhynchos domestica*) CCK1R gene sequence was appended with restriction enzyme (EcoRI/XhoI) cut site and protective bases, primers were re-designed, and PCR amplification was carried out. PCR reaction was re-run as above, but at an annealing temperature of 64 °C. PCR primers used were as follows: forward primer 5′-CCGGAATTCATGGAAATAGTTGAAG-3′ and reverse primer 5′-GCTCTAGATCAGGGGGGTGCAGAT-3′. Agarose gel verification, product recovery, T-vector ligation, transformation, amplification on resistant medium, PCR verification, and sequencing were performed. The verified sequence was uploaded to the NCBI database (accession number MN250295.1).

### 2.4. Immunocytochemistry

The freshly isolated rat, mouse, and Peking duck pancreatic acini were fixed in 4% paraformaldehyde in phosphate buffered saline (PBS, 10 min) after attachment to Cell-Tak-coated cover-slips. Fixed pancreatic acini were permeabilized in 0.2% Triton X-100 (in PBS) for 15 min. Non-specific binding was blocked in 3% BSA (in PBS) before incubation with goat anti-human CCK1R primary antibody (dilution 1:100) in a wet chamber at 4 °C overnight, followed by incubation with TRITC-conjugated donkey anti-goat secondary antibody (dilution 1:100) for 30 min. The attached pancreatic acini were washed in PBS before incubation with primary antibody, and thereafter in PBS containing 0.2% Triton X-100 and 2% Tween-20. Cover-slips were placed on glass slides, sealed, and stored at 4 °C in the dark before confocal imaging (Zeiss LSM 510 META) under objective 63×/1.40 oil, with λ_ex_ 543 nm.

### 2.5. Photodynamic Treatment

Rat, mouse, or Peking duck pancreatic acini, attached to the bottom Cell-Tak-coated cover-slip of a Sykes–Moore perfusion chamber, were perifused in Krebs buffer, but without glutamine, BSA, amino acid mixture or trypsin inhibitor. SALPC (1 or 2 μM) was added to the perfusion buffer and LED 675 nm light (LAMPLIC, Shenzhen, China) was applied as indicated in the calcium tracings at a power density of 40, 50, or 60 mW·cm^−2^ for 1.5 min. LED light power density was measured at the level of attached acini in the Sykes–Moore chamber with a power meter (IL1700, Intl.Light Inc., Newburyport, MA, USA).

The optimized photodynamic intensities as indicated above were determined in preliminary experiments, where different photodynamic intensities were used in isolated rat, mouse, and Peking duck pancreatic acini. For light irradiation of SALPC-treated pancreatic acini, red light (>580 nm) from a halogen cold light source (Hoya-Schott, HL100R, Tokyo, Japan), red LED 650 nm, and red LED 675 nm (LAMPLIC, Shenzhen, China) were all tested. Data obtained in preliminary experiments are not shown. Data in Results were obtained with LED 675 nm.

### 2.6. Calcium Imaging

The freshly isolated rat, mouse, or Peking duck pancreatic acini were loaded with Fura-2 AM (10 µM) in a shaking water-bath (37 °C, 30 min, 50 cycles per min). Fura-2-loaded pancreatic acini were attached to the bottom cover-slip (coated with Cell-Tak, 1.7 g·L^−1^, 3 µL on each cover-slip) of Sykes–Moore perfusion chambers.

The perfusion chamber was placed on the platform of a Nikon NE 3000 inverted fluorescence microscope connected to a calcium imaging device (Photon Technology International, PTI, Edison, NJ, USA) with alternating excitations at 340 nm/380 nm (PTI monochromator DeltaRam X). Emission (emitter 510 ± 40 nm) was detected with a CCD (NEO-5.5-CL-3, Andor/Oxford Instruments, Belfast, UK). Fluorescence ratios F_340_/F_380_ indicative of cytosolic calcium concentration were plotted against time, as reported previously [14,15,19,20,24].

### 2.7. Statistical Analysis

All data are presented as mean ± SEM (standard error of means). A Student’s *T*-test was performed, and a statistically significant difference at *p* < 0.05 is indicated by an asterisk (*).

## 3. Results

### 3.1. Photodynamic CCK1R Activation in Rat Pancreatic Acini

**Figure 1:**
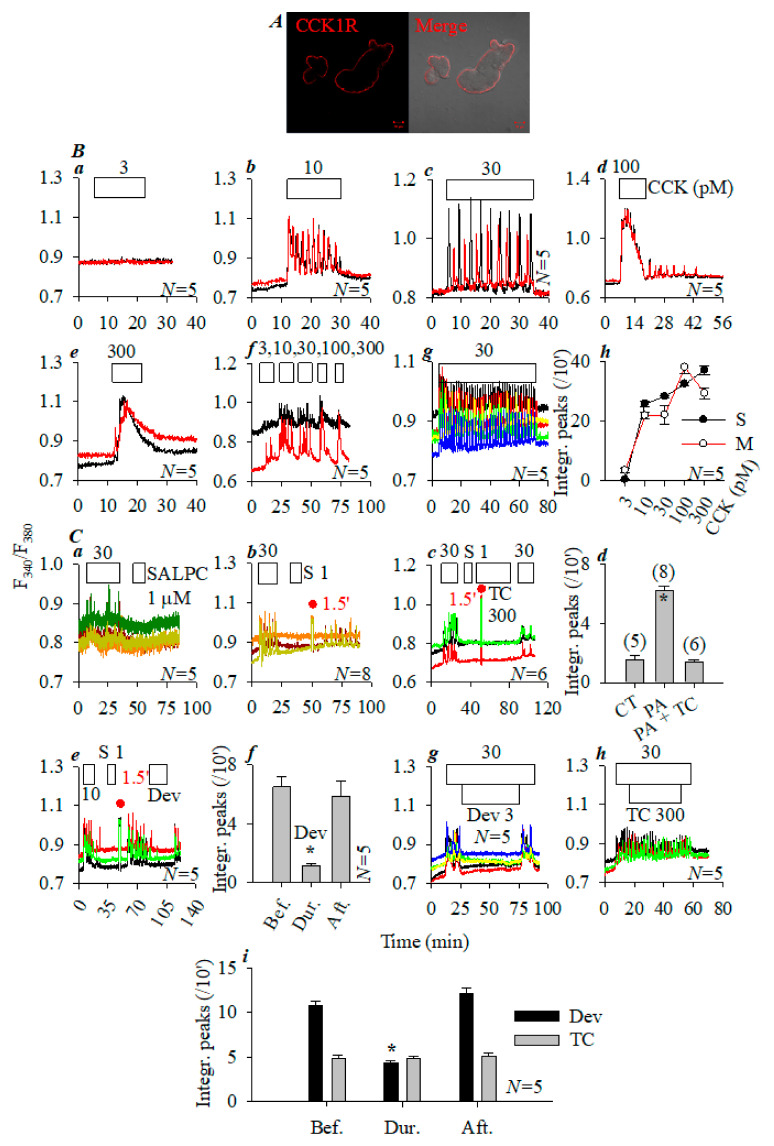
**Photodynamic CCK1R activation in rat pancreatic acini.** Pancreatic acini isolated from Sprague-Dawley (SD) rat were fixed, incubated with goat anti-human CCK1R^242−257^ antipeptide primary antibody and TRITC-conjugated donkey anti-goat secondary antibody, and confocal imaged; fluorescent (red) and merged (with bright-field) images are shown (**A**). Confocal images were taken in a Zeiss LSM 510 META (objective 63×/1.40 oil) with λ_ex_ 543 nm. Scale bars: 10 μm. Pancreatic acini loaded with Fura-2 AM were attached to the bottom cover-slip of the Sykes–Moore perfusion chamber and perifused; CCK 3 (**Ba**),10 (**Bb**), 30 (**Bc**), 100 (**Bd**), and 300 pM (**Be**) were added as indicated. CCK 3, 10, 30, 100, and 300 pM were also added sequentially to the same pancreatic acini (**Bf**), or CCK 30 pM was perfused over an extended time period (**Bg**). (**Bh**) Integrated calcium peak areas above the baseline in calcium tracings as shown in (**Ba**–**Be**) were calculated (10–25 min,) from multiple repeat experiments (*N* = 5); statistical data are presented (**Bh**) (black curve, S for single dose). Integrated calcium peaks were similarly calculated for sequential CCK doses (M for multiple doses added to the same pancreatic acini) from calcium tracings shown in (**Bf**) from repeat experiments (each dose for 10 min: 5–15, 20–30, 35–45, 50–60, and 65–75 min); statistical data are presented (**Bh**) (red curve, M for multiple doses). CCK 30 pM, SALPC 1 μM, Trolox C 300 μM, and LED light (675 nm, 60 mW·cm^−2^, 1.5 min) were applied as indicated in (**Ca**–**Cc**). (**Ca**) CCK 30 pM, SALPC 1 μM. (**Cb**) CCK 30 pM, SALPC 1 μM, and LED light (1.5 min). (**Cc**) CCK 30 pM, SALPC 1 μM, Trolox C 300 μM, LED light (1.5 min), and CCK 30 pM. Integrated calcium peak areas (55–80 min, per 10 min) above the baseline in typical calcium tracings shown in (**Ca**–**Cc**) were calculated from multiple repeat experiments (*N* = 5–8); statistical data are presented (**Cd**). CT, control; PA, photodynamic action. (**Ce**) CCK 10 pM, SALPC 1 μM, LED light (1.5 min), devazepide 3 nM. Integrated calcium peak areas above the baseline (per 10 min) before, during, and after devazepide addition as shown in (**Ce**) was calculated from multiple repeat experiments; statistical data are presented (**Cf**). (**Cg**) CCK 30 pM, devazepide (Dev) 3 nM. (**Ch**) CCK 30 pM, Trolox C (TC) 300 μM. Integrated calcium peaks above the baseline before, during, and after devazepide or Trolox C (per 10 min) in calcium tracings shown in (**Cg**,**Ch**) were calculated from repeat experiments (*N* = 5) and are presented (**Ci**). Student’s *T*-test was used; an asterisk (*) in (**Cd**, **Cf**, **Ci**) indicates statistical significance at *p* < 0.05 (*N* = 5–8). The abscissa in panels (**Ba**–**Bg**,**Ca**–**Cc**,**Ce**,**Cg**,**Ch**) are time in min, as indicated.

Rat pancreatic acini were fixed for immunocytochemistry, which revealed basolateral plasma membrane localization of CCK1R (Figure 1A). The freshly isolated rat pancreatic acini were perifused and CCK was added to the perifusion buffer. CCK 3 pM was without any effect (Figure 1Ba), CCK 10, 30 pM induced regular calcium oscillations (Figure 1Bb,Bc), whereas CCK 100 pM elicited a phasic increase followed by sparse calcium spikes upon washout (Figure 1Bd). CCK 300 pM induced phasic increases, followed by a plateau after wash out (Figure 1Be). Sequential CCK concentrations (3, 10, 30, 100, and 300 pM) demonstrated a similar dose response relationship (Figure 1Bf). Continued CCK 30 pM triggered regular calcium oscillations over an extended period (70 min, Figure 1Bg). Statistical analysis revealed monophasic dose–response curves, with single (S) or sequential multiple (M) doses (Figure 1Bh).

After CCK-induced calcium spikes were washed out, the addition of photosensitizer sulphonated aluminum phthalocyanine (SALPC) 1 µM alone had no effect (Figure 1Ca). However, if after sequential CCK 30 pM/SALPC 1 µM doses, a brief red LED light pulse (675 nm, 60 mW·cm^−2^, 1.5 min) was applied, persistent calcium oscillations appeared, which lasted to the end of the experiment (Figure 1Cb). If photodynamic action (SALPC 1 µM; light 675 nm, 60 mW·cm^−2^, 1.5 min) was triggered in the presence of ^1^O_2_ quencher Trolox C 300 µM, the LED light pulse no longer induced any calcium increases (Figure 1Cc). Statistical analysis of data from multiple experiments as demonstrated in Figure 1Ca–Cc confirmed that SALPC alone had no effect (Figure 1Ca), photodynamic action (SALPC 1 µM; LED 675 nm, 60 mW·cm^−2^, 1.5 min) induced calcium increases (Figure 1Cb), and those calcium increases were blocked if photodynamic action (SALPC 1 µM; LED 675 nm, 60 mW·cm^−2^, 1.5 min) was triggered in the presence of Trolox C (Figure 1Cd, *p* < 0.05). SALPC photodynamic action thus triggered calcium oscillations in rat pancreatic acini, which were blocked by the simultaneous presence of Trolox C.

Photodynamic action (SALPC 1 µM; light 675 nm, 60 mW·cm^−2^, 1.5 min)-triggered calcium oscillations persisted after LED light irradiation. Such persistent calcium oscillations were blocked reversibly by devazepide 3 nM (Figure 1Ce). The reversible inhibition was statistically significant (Figure 1Cf, *p* < 0.05).

While calcium oscillations induced by CCK 30 pM were inhibited by devazepide 3 nM (Figure 1Cg), ^1^O_2_ quencher Trolox C 300 µM had no effect (Figure 1Ch). Statistical analysis confirmed devazepide inhibition, but the lack of effect of Trolox C (Figure 1Ci).

These experiments with rat pancreatic acini were repeated with mouse and Peking duck pancreatic acini for comparison.

### 3.2. Photodynamic CCK1R Activation in Mouse Pancreatic Acini

The experimental data obtained from mouse pancreatic acini are presented as Figure S1 in the Supplementary Materials.

### 3.3. Photodynamic CCK1R Activation in Peking Duck Pancreatic Acini

**Figure 2:**
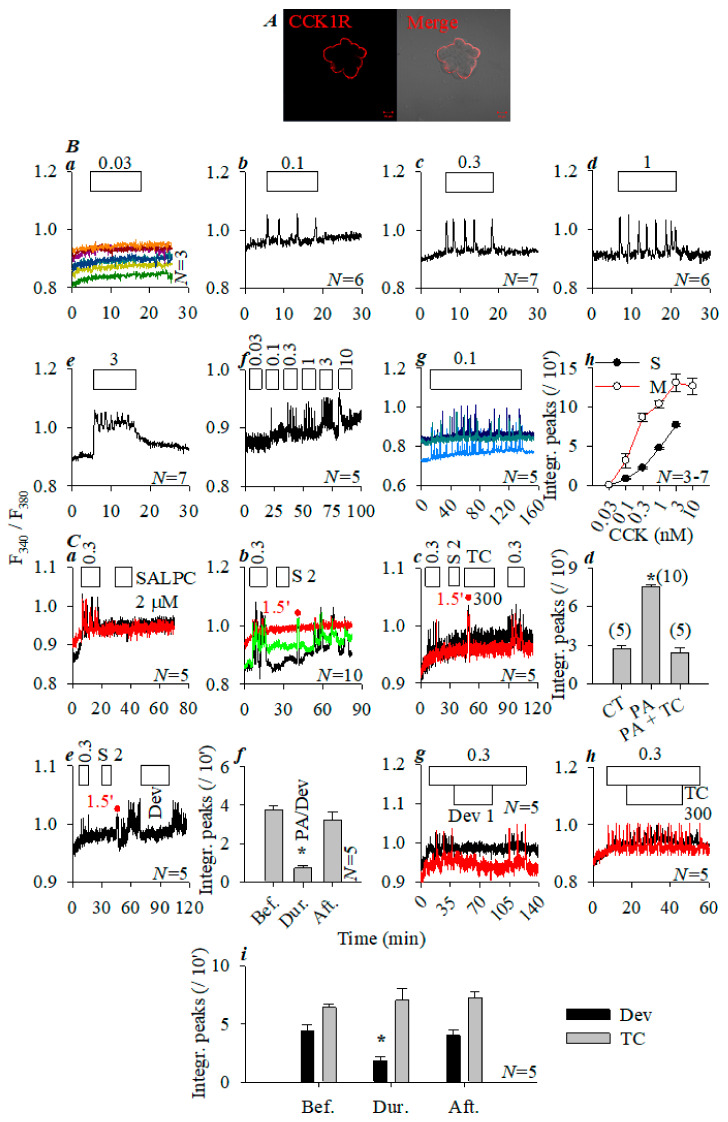
**Photodynamic CCK1R activation in duck pancreatic acini.** Peking duck (*Anas platyrhynchos domestica*) pancreatic acini were processed for immunocytochemistry (primary goat anti-human CCK1R^242−257^ antibody and secondary TRITC-conjugated donkey anti-goat antibody) and confocal imaged (**A**) in a Zeiss LSM 510 META (objective 63 ×/1.40 oil) with λ_ex_ 543 nm: fluorescent (red) and merged (with bright field image) images. Scale bars: 10 µm. Duck pancreatic acini loaded with Fura-2 AM were perifused; CCK 0.03 (**Ba**), 0.1 (**Bb**), 0.3 (**Bc**), 1 (**Bd**), and 3 nM (**Be**) were added in different pancreatic acini separately; or CCK 0.03, 0.1, 0.3, 1, 3, and 10 nM were added in the same acini sequentially (**Bf**). Continued exposure to CCK 100 pM (**Bg**). Calcium peak areas above the baseline in original tracings represented by (**Ba**–**Be**) (from 5–20 min, black) and (**Bf**) (5–15, 20–30, 35–45, 50–60, 65–75, and 80–90 min) were calculated from multiple experiments. Dose response curves for single (S) or sequential multiple doses (M) are shown (**Bh**, N = 3–7). CCK, SALPC, devazepide (Dev), Trolox C (TC), and LED light irradiation (675 nm, 40 mW·cm^−2^, 1.5 min) were applied as indicated. (**Ca**) CCK 0.3 nM, SALPC 2 µM. (**Cb**) CCK 0.3 nM, SALPC 2 µM, and LED light. (**Cc**) CCK 0.3 nM, SALPC 2 µM, Trolox C (TC) 300 µM, LED light, and CCK 0.3 nM. The calcium peak area above the baseline in the original calcium tracings in multiple identical experiments, but represented by (**Ca**–**Cc**), was calculated and shown as integrated calcium peaks per 10 min (**Cd**). (**Ce**) CCK 0.3 nM, SALPC 2 µM, LED light (1.5 min), and devazepide (Dev) 1 µM. The typical experiment shown in (**Ce**) was repeated multiple times (*N* = 5) and integrated calcium peak areas above the baseline before, during, and after devazepide (Dev) were calculated (per 10 min); statistical data were presented (**Cf**). (**Cg**) CCK 0.3 nM; devazepide (Dev) 1 µM. (**Ch**) CCK 0.3 nM; Trolox C (TC) 300 µM. The experiments shown in (**Cg**,**Ch**) were repeated multiple times (*N* = 5); calcium peak areas above the baseline (per 10 min) before, during, and after devazepide or Trolox C were calculated and the statistical data are presented in (**Ci**). For all statistical analyses shown in (**Bh**,**Cd**,**Cf**,**Ci**), Student’s *T*-test was performed, with the asterisk (*) indicating statistical significance at *p* < 0.05 (N = 3–7, 5–10, 5, and 5, respectively). The abscissa in panels (**Ba**–**Bg**,**Ca**–**Cc**,**Ce**,**Cg**,**Ch**) are time in min, as indicated.

Peking duck (*Anas platyrhynchos domestica*) ducklings (250–450 g) are suitable to isolate pancreatic acini from the spleen lobe. Immunocytochemistry confirmed basolateral CCK1R distribution in Peking duck pancreatic acini (Figure 2A). In perifused Peking duck pancreatic acini, CCK 0.03 nM (i.e., 30 pM) had no effect (Figure 2Ba); CCK 0.1, 0.3, and 1 nM induced calcium spikes of increasing frequency (Figure 2Bb–Bd); and CCK 3 nM elicited calcium oscillations superimposed on a plateau (Figure 2Be). Sequential addition of CCK 0.03, 0.1, 0.3, 1, 3, and 10 nM induced dose-dependent calcium responses (Figure 2Bf). Extended perifusion with CCK 100 pM triggered regular calcium oscillations (Figure 2Bg). Similar dose–response (Figure 2Bh) was seen with CCK added to different pancreatic acini (Figure 2Ba–Be) or added sequentially to the same pancreatic acini (Figure 2Bf).

After the calcium oscillations induced by CCK 300 pM were washed out, the addition of photosensitizer SALPC 2 µM alone had no effect (Figure 2Ca). Following sequential CCK 300 pM/SALPC 2 µM, red LED light (675 nm, 40 mW·cm^−2^, 1.5 min) triggered robust fresh calcium spikes (Figure 2Cb). However, if photodynamic action (SALPC 2 µM; LED 675 nm, 40 mW·cm^−2^, 1.5 min) was repeated in the presence of Trolox C 300 µM, LED light irradiation no longer induced any calcium increases (Figure 2Cc). Statistical analysis confirmed blockade of photodynamic action (SALPC 2 µM; LED 675 nm, 40 mW·cm^−2^, 1.5 min) by Trolox C 300 µM (*p* < 0.05, Figure 2Cd).

SALPC photodynamic action (SALPC 2 μM; LED 675 nm, 40 mW.cm^−2^, 1.5 min)-triggered calcium oscillations in Peking duck pancreatic acini were inhibited reversibly by CCK1R antagonist devazepide 1 µM (Figure 2Ce). Statistical analysis of data from multiple repeat experiments, as shown in Figure 2Ce, corroborated that devazepide inhibition was significant (*p* < 0.05, Figure 2Cf). In parallel control experiments, it was observed that CCK 300 pM-induced calcium oscillations were inhibited by devazepide 1 µM (Figure 2Cg), but Trolox C 300 μM has no effect (Figure 2Ch), and devazepide inhibition was statistically significant (*p* < 0.05, Figure 2Ci).

Basolaterally localized Peking duck pancreatic acinar cell CCK1R was thus photodynamically activated, likely via oxidative reactions of singlet oxygen.

### 3.4. Comparison of Photodynamic Activation of Rat, Mouse, and Peking Duck Pancreatic Acinar Cell CCK1R

Picomolar CCK (10, 30 pM) induced regular calcium oscillations in rat and mouse pancreatic acini (Figure 1Bb,Bc,Bg and Figure S1Bb), but high picomolar to nanomolar CCK (0.1, 0.3, 1 nM) was required to induce regular calcium oscillations in Peking duck pancreatic acini (Figure 2Bb–Bd,Bg). The CCK concentration needed to induce regular calcium oscillations, as defined in the literature [25,26], was 30 pM, 10 pM, and 300 pM in rat, mouse, and Peking pancreatic acini, respectively, with a concentration ratio of 3:1:30 (Table 1).

Photodynamic action triggered robust calcium oscillations in rat, mouse, and Peking duck pancreatic acini (Figure 1Cb, Figure 2Cb and Figure S1Cb) (Table 1). The rat:mouse:Peking duck photodynamic intensity ratio was 60:50:80 μM × mW·cm^−2^ (1.2:1:1.6). The ratio of agonist (CCK) concentration (3:1:30) was more wide-ranging than photodynamic intensity (1.2:1.0:1.6). It may be noted here that, for photodynamic activation, lower than optimal photodynamic intensity induced no or only sparse calcium spikes and higher intensities induced a calcium plateau (data not shown).

Although some difference is noted in both agonist-stimulated and photodynamic activation of rat, mouse, and Peking duck pancreatic acinar cell CCK1R, it is now confirmed that mouse and Peking duck pancreatic acinar cell CCK1R could be photodynamically activated, as rat pancreatic acinar cell CCK1R (this work and [12]) and human CCK1R ectopically expressed in CHO and HEK293 cell lines could be [13,14,15,19,20,21].

### 3.5. Structural Basis of Photodynamic Activation of Rat, Mouse, and Peking Duck CCK1R

**Figure 3:**
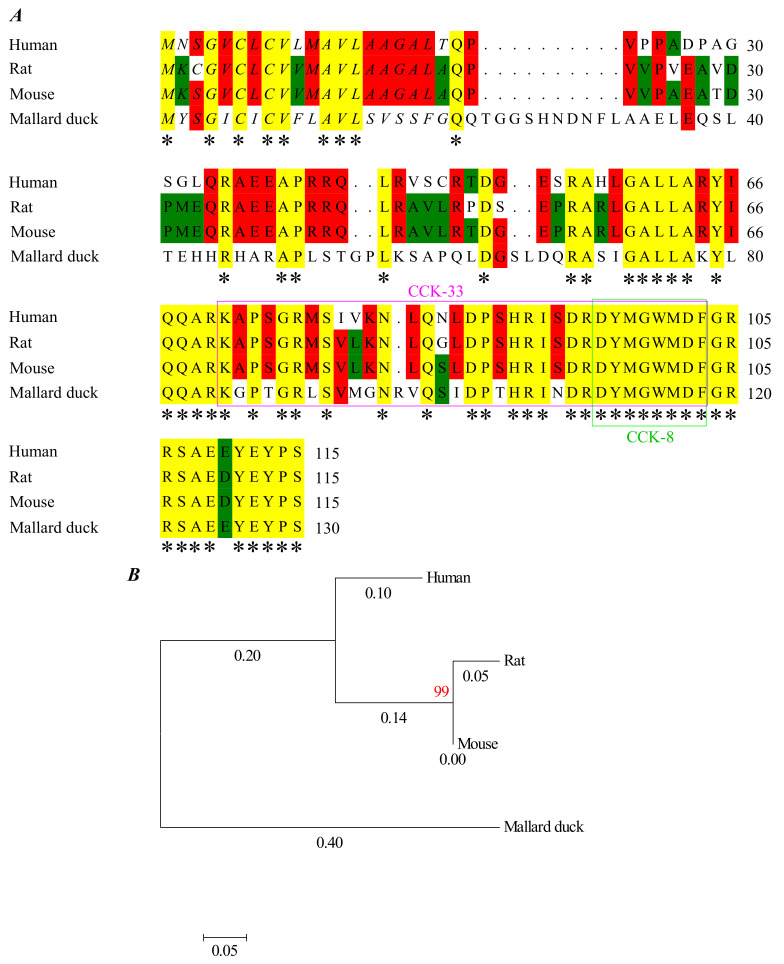
**Human, rat, mouse, and mallard duck preproCCK.** (**A**) Aligned peptide sequences of human (NP_000720.1), rat (NP_036961.1), mouse (XP_017168605.1), and mallard duck (XP_038031411.1) preproCCK. Alignment made by Mega 7.0 using clustalW. Annotations: asterisks (*) (yellow background), conserved residues; *italics*, signal peptide sequence; pink box, CCK-33; green box, CCK-8. (**B**) Phylogenetic tree drawn by Mega 7.0 using the maximum likelihood method for 1000 calculations. Red number, confidence. Black numbers, evolutionary distance.

At first glance, varied efficacy of CCK-stimulated activation of rat, mouse, and Peking duck pancreatic acini might be due to peptide sequence variation in CCK. A comparison of rat (NP_036961.1), mouse (XP_017168605.1), and mallard duck (XP_038031411.1) (a close relative to Peking duck) and human (NP_000720.1) prepro-CCK peptide showed a sequence homology of 78%, 80%, and 53%, respectively (Figure 3). The intermediate–long human CCK-33 peptide showed a sequence homology with rat, mouse, and mallard duck CCK-33 of 91%, 91%, and 67%, respectively (Figure 3). The human, rat, mouse, and duck sulphated CCK-8 octapeptide (used in this work), however, are completely identical (Figure 3). The CCK concentration difference to activate CCK1R in rat, mouse, and Peking duck pancreatic acini (3:1:30) to induce regular calcium oscillations, as defined in [25,26] (Figure 1, Figure 2 and Figure S1; Table 1), must rest with the target receptor CCK1R, instead of the sulphated CCK octapeptide.

To compare the complete protein sequence of rat, mouse, Peking duck CCK1R, we first need to obtain the Peking duck CCK1R protein sequence, which was not present in any database previously, but a CCK1R sequence of the related mallard duck was found (XP_005019307.1).

**Figure 4:**
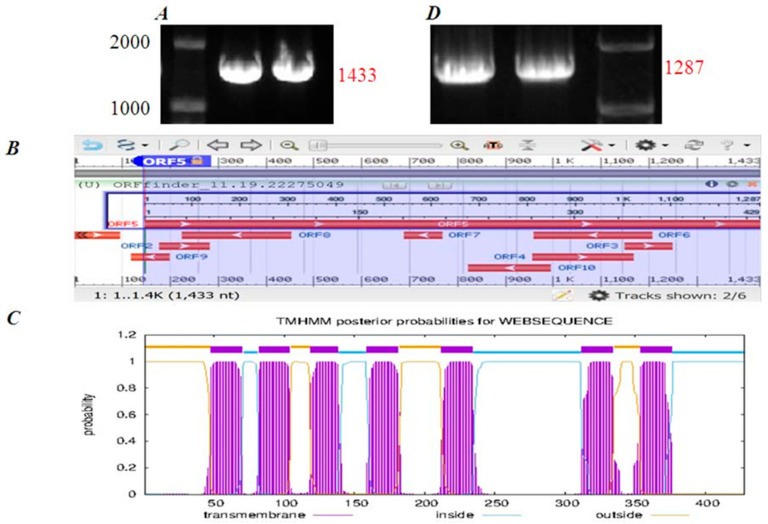
**Cloning of Peking duck CCK1R gene.** (**A**) RT-PCR product 1433 bp from mallard duck (*Anas platyrhynchos*). (**B**) Open reading frames in the sequence of RT-PCR product as analyzed by ORF Finder. (**C**) Transmembrane topology of ORF5 as analyzed by TMHMM. (**D**) Bands marked RT-PCR product 1287 bp from Peking duck (*Anas platyrhynchos domestica*).

A DNA fragment of 1433 bp was cloned by RT-PCR from isolated Peking duck pancreatic acini, with primers derived from the mallard duck (*Anas platyrhynchos*) CCK1R gene (XP_005019307.1) (Figure 4A). Target band was recovered and attached to T-vector. Ligation products were transformed into *E. coli* (Top10) and resistant strains screened on an ampicillin-resistant LB plate. Resistant strains were expanded in LB liquid medium + ampicillin for DNA sequencing. The sequence obtained (Sangon Biotech, Beijing, China) was analyzed with Open Reading Frame (ORF) Finder (www.ncbi.nlm.nih.gov/gorf/gorf.html, accessed on 19 November 2022). Ten ORFs were identified from the cloned gene sequence (1433 bp): five in the forward strand (1–96, 178–282, 1150–1248, 956–1168, 147–1433) and five in the reverse strand (1207–959, 769–689, 454–224, 199–119, 996–823) (Figure 4B). The ORFs were subject to TMHMM2.0 (www.cbs.dtu.dk/services/TMHMM/, accessed on 19 November 2022) analysis, to predict transmembrane topology; only 147–1433 was found to encode a seven-transmembrane-domain protein (Figure 4C). This ORF5 (147–1433, 1287 bp) is thus concluded to be the CCK1R sequence of Peking duck (*Anas platyrhynchos domestica*) pancreatic acini. New primers for RT-PCR were designed from ORF 147-1433 to amplify the Peking duck CCK1R gene (Figure 4D), which was then registered at the NCBI database (MN250295.1).

**Figure 5:**
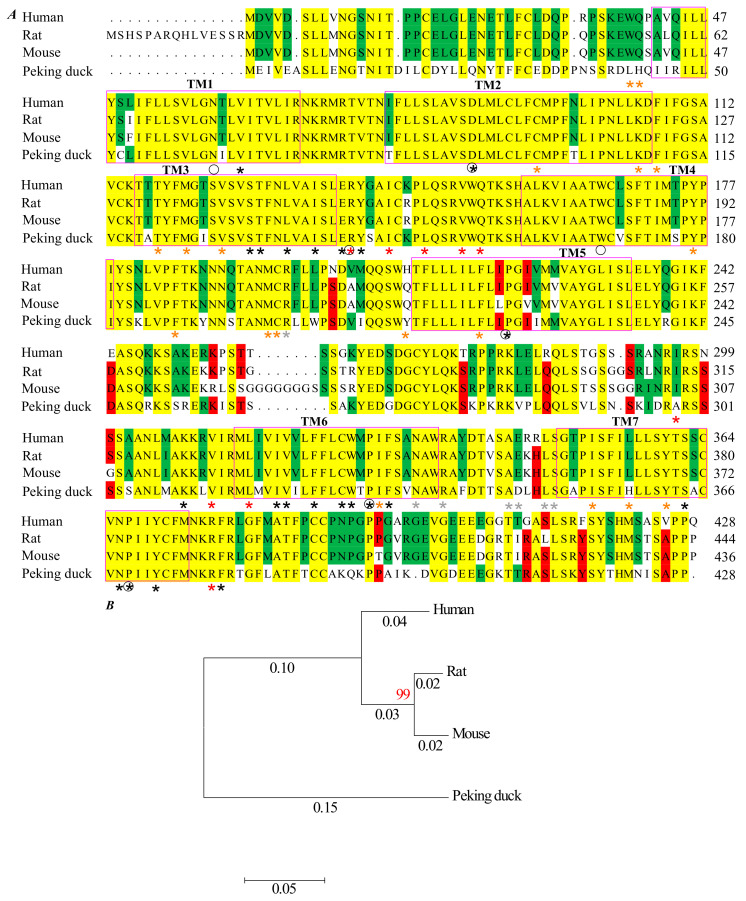
**Aligned protein sequence of human, rat, mouse, and Peking duck CCK1R.** (**A**) Human (NP_000721.1), rat (NP_036820.1), mouse (AAH20534.1), and Peking duck sequences (MN250295.1 for nulceotide sequence, QIJ58473.1 for protein sequence) are aligned by Mega 7.0 using clustalW. TM1–7 are outlined by pink rectangles. Asterisks (*) indicate key residues: grey, CCK-8 recognition; brown, CCK-8 binding; black, CCK1R activation; red, G protein-binding. Black circles indicate Ballesteros–Weinstein [30] reference residues N^1.50^, D^2.50^, R^3.50^, W^4.50^, P^5.50^, P^6.50^, and P^7.50^. (**B**) Phylogenetic tree generated from Mega 7.0 via the maximum likelihood method for 1000 calculations. Red numbers, confidence. Black numbers, evolutionary distance.

Probably unsurprisingly, the deduced Peking duck CCK1R protein sequence was found to be completely identical to mallard duck CCK1R, both being 428 residues long, thus only the Peking duck sequence is shown in Figure 5 for inter-species protein sequence comparison. Although the Peking duck CCK1R protein sequence was found to be identical to the reported mallard duck CCK1R protein sequence, the coding sequences of these two were found to differ slightly. For CCK1R residues Asp^17^, Leu^46^, Val^128^, Ala^161^, Ser^262^, and Leu^384^, the codons in the Peking duck sequence were GAC, CTA, GTC, GCG, AGT, and CTT, but in mallard duck, the corresponding codons were GAT, CTG, GTA, GCC, AGC, and CTG. Note the single nucleotide difference in each codon.

The human, rat, mouse, and Peking duck CCK1R protein sequences were then compared. The rat (NP_036820.1), mouse (AAH20534.1), and Peking duck CCK1R (MN250295.1 for nucleotide sequence, QIJ58473.1 for protein sequence) showed a sequence homology of 91%, 91%, and 75%), respectively, with the human CCK1R (NP_000721.1) respectively. In the transmembrane domains 1–7 (TM1–7), the sequence homology increased to 98%, 97%, and 89%, respectively (Figure 5A). The phylogenetic tree shows the relationship of human, rat, mouse, and Peking duck CCK1R protein sequences (Figure 5B).

Early point mutation experiments with human CCK1R identified residues important for CCK recognition, binding, and CCK1R receptor activation (W^39/1.30^, Q^40/1.31^, C^94/2.57^, F^107/ECL1^, M^121/3.32^, V^125/3.36^, M^195/ECL2^, R^197/ECL2^, F^218/5.47^, W^326/6.48^, I^329/6.51^, F^330/6.52^, N^333/6.55^, R^336/6.58^, I^352/7.35^, L^356/7.39^, Y^360/7.43^, N^366/7.49^) [27,28,29] (Figure 5A; Table 2). The examination of aligned CCK1R sequences (Figure 5A) found that most of these residues in human CCK1R are conserved in rat and mouse CCK1R, but W^39/1.30^, Q^40/1.31^, and L^356/7.39^ in human CCK1R were found to be changed to L^39/1.30^, H^40/1.31^, and H^358/7.39^ in Peking duck CCK1R (Figure 5A; Table 2). This might account, at least partially, for the decreased CCK affinity in Peking duck pancreatic acinar cell CCK1R (Figure 1, Figure 2 and Figure S1; Table 1), as W^39/1.30^ and Q^40/1.31^ mutations each decreased CCK binding affinity 10-fold, while L^356/7.39^ mutation decreased CCK binding affinity 8-fold [28].

The point mutation experiments mentioned above were corroborated by the more recently solved complex structure of human CCK1R bound to sulphated CCK octapeptide. In the human CCK1R structure, a CCK-binding pocket was identified (composed of K^105/ECL1^, F^107/ECL1^, T^118/3.29^, M^121/3.32^, Y^176/4.60^, F^185/ECL2^, M^195/ECL2^, C^196/ECL2^, R^197/ECL2^, H^207/5.39^, N^333/6.55^, R^336/6.58^, A^343/ECL3^, E^344/ECL3^, L^347/ECL3^, S^348/ECL3^, I^352/7.35^, and Y^360/7.43^) [31] (Table 2). Again, these residues are largely conserved in rat and mouse CCK1R (Figure 5A; Table 2). Although E^344/ECL3^ in human CCK1R remained the same in rat (E^360/ECL3^) and mouse (E^352/ECL3^) CCK1R, the corresponding Peking duck residue was D^346/ECL3^ instead (Figure 5A; Table 2). In addition, the corresponding H^207/5.39^ in human CCK1R was found to be Q^222/5.39^ in rat, Q^207/5.39^ in mouse, but Y^210/5.39^ in Peking duck CCK1R (Figure 5A; Table 2). This double mutation (human E^344/ECL3^, rat E^360/ECL3^, mouse E^352/ECL3^, to Peking duck D^346/ECL3^; human H^207/5.39^, rat Q^222/5.39^, mouse Q^207/5.39^, to Peking duck Y^210/5.39^) (Table 2) in the CCK-binding pocket will contribute to the decreased CCK sensitivity of Peking duck CCK1R (Figure 1, Figure 2, and Figure S1; Table 1), although it is not known at the present time why the mouse CCK1R was more sensitive to CCK stimulation than the rat CCK1R (Table 1).

It is known that agonist binding to class A members of GPCR triggers a series of layered transitions (from extracellular to intracellular) of receptor protein structure conformation [32]. More specifically, for CCK binding to human CCK1R, these layered changes were also defined, involving these bracketed residues (V^62/1.53^, D^87/2.50^, L^133/3.43^, I^136/3.46^, R^139/3.50^, K^308/6.30^, I^318/6.40^, V^319/6.41^, F^322/6.44^, W^326/6.48^, F^330/6.52^, S^362/7.45^, N^366/7.49^, Y^370/7.53^, and F^377/8.50^), with eventual binding and activation of Gq protein [33]. Remarkably, residues involved in layered activation signal transitions are completely identical among human, rat, mouse, and Peking duck CCK1R (Figure 5A, Table 2).

Activated human CCK1R in dynamics modeling was found to bind Gq by hydrophobic interactions of cytosolic facing residues (I^143/3.54^, I^296/ICL3^, V^311/6.33^, and L^315/6.37^) [31], by salt bridge-formation (R^376/8.49^) [31], and by hydrogen bonding of cytosolic cavity-forming residues (R^139/3.50^, L^147/ICL2^, V^151/ICL2^, and Q^153/ICL2^) [34]. Most of these residues are identical in human (R^139/3.50^, I^143/3.54^, L^147/ICL2^, V^151/ICL2^, Q^153/ICL2^, V^311/6.33^, L^315/6.37^, and R^376/8.49^), rat (R^154/3.50^, I^158/3.54^, L^162/ICL2^, V^166/ICL2^, Q^168/ICL2^, V^327/6.33^, L^331/6.37^, and R^392/8.49^), mouse (R^139/3.50^, I^143/3.54^, L^147/ICL2^, V^151/ICL2^, Q^153/ICL2^, V^319/6.33^, L^323/6.37^, and R^384/8.49^), and Peking duck (R^142/3.50^, I^146/3.54^, L^150/ICL2^, V^154/ICL2^, Q^156/ICL2^, V^313/6.33^, L^317/6.37^, and R^378/8.49^) CCK1R, but human I^296/ICL3^ (hydrophobic index 4.5), rat I^312/ICL3^, and mouse I^304/ICL3^ were found to correspond to Peking duck residue A^298/ICL3^ (hydrophobic index of 1.8) (Figure 5A). This is likely correlated to decreased efficiency in Gq coupling, either with CCK stimulated and photodynamic activation of Peking duck pancreatic acinar cell CCK1R (Figure 1, Figure 2 and Figure S1; Table 1), because it is known that I^296/ICL3^ (hydrophobic index 4.5) mutation to G (hydrophobicity −0.4) significantly weakens human CCK1R coupling to Gq [31].

To summarize, residues to recognize and bind sulphated CCK octapeptide are mostly conserved in human, rat, mouse, and Peking duck CCK1R, but with the following exceptions: human W^39/1.30^, Q^40/1.31^, H^207/5.39^ (rat Q^222/5.39^, mouse Q^207/5.39^), E^344/ECL3^, and L^356/7.39^ are replaced in Peking duck by L^39/1.30^, H^40/1.31^, Y^210/5.39^, D^346/ECL3^, and H^358/7.39^, respectively (Table 2). Residues involved in transmembrane conduction of CCK1R layered conformational changes are completely identical in human, rat, mouse, and Peking duck CCK1R (Table 2). Residues involved in transduction of activation information from CCK1R to Gq protein are mostly conserved, but the human residue I^296/ICL3^ (rat I^312/ICL3^ and mouse I^304/ICL3^) is replaced by A^298/ICL3^ in Peking duck CCK1R (Table 2). The variation in residues for CCK recognition, binding, and G protein coupling, but no difference for layered transmembrane (transmembrane domains) conduction of receptor activation signaling, could account for reduced efficacy of CCK stimulated CCK1R activation in isolated Peking duck pancreatic acini when compared with rat and mouse pancreatic acini (Figure 1, Figure 2 and Figure S1; Table 1).

Met (M), Trp (W), Cys (C), His (H), and Tyr (Y) residues are recognized targets of ^1^O_2_ oxidation in type II photodynamic action [35,36]. Out of the key residues in CCK-stimulated CCK1R activation described above, the following 10 are susceptible to ^1^O_2_ oxidation: C^94/2.57^, M^121/3.32^, Y^141/3.51^, Y^176/4.60^, M^195/ECL2^, C^196/ECL2^, H^207/5.39^, W^326/6.48^, Y^360/7.43^, and Y^370/7.53^. Of these residues, Y^141/3.51^ and Y^370/7.53^ in motifs of E^3.49^R^3.50^Y^3.51^ and N^7.49^P^7.50^xxY^7.53^, respectively, are known to be involved in G protein binding in all class A GPCR [32], Y^370/7.53^ is also involved in the layered transmembrane signal conduction [33] (Figure 5A; Table 2). Out of these 10 residues, 9 are completely identical in human, rat, mouse, and Peking duck CCK1R; but human H^207/5.39^ is changed to rat Q^222/5.39^, mouse Q^207.5.39^, and Peking duck Y^210/5.39^ (Figure 5A, Table 2). This might account for the conserved pharmacological property of photodynamic activation of rat, mouse, and Peking duck pancreatic acinar cell CCK1R (Figure 1, Figure 2 and Figure S1; Table 1). The variation in the potency of photodynamic activation of rat, mouse, and Peking duck CCK1R (mouse > rat > Peking duck) might be related to point mutation at position^5.39^ located in the CCK octapeptide-binding pocket (human H^207/5.39^, rat Q^222/5.39^, mouse Q^207/5.39^, and Peking duck Y^210/5.39^). The change in Gq protein-binding residue of human I^296/ICL3^ (rat I^312/ICL3^, mouse I^304/ICL3^, to Peking duck A^298/ICL3^ (hydrophobic index of 1.8) (Figure 5A, Table 2) could be accountable too, but isoleucine is not susceptible to ^1^O_2_ oxidation.

**Figure 6:**
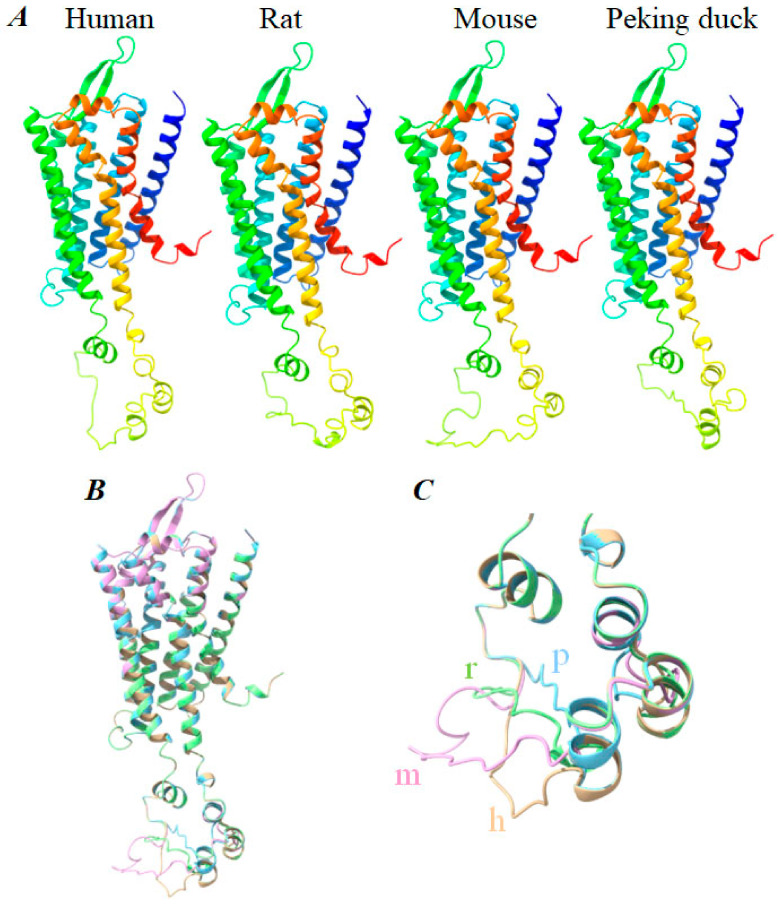
**Comparison of human, rat, mouse, and Peking duck CCK1R structure.** The 3D structure of human, rat, mouse, and Peking duck CCK1R structures are generated by Swiss-Model using human CCK1R (PDB: 7F8X) as the template. (**A**) Human, rat, mouse, and Peking duck CCK1R, each in color gradient (from blue to red) from N-terminus to C-terminus. (**B**) Human, rat, mouse, and Peking duck CCK1R structures are merged with ChimeraX 1.3. Color code: human, brown; rat, green; mouse, pink; and Peking duck blue. (**C**) Expanded view of the intracellular loop 3 (ICL3) of human, rat, mouse, and Peking duck CCK1R superimposed, color coded as in (**B**). Labeling on structure: h, human; r, rat; m, mouse; p, Peking duck. Note the disordered regions in mouse (A^250^–K^286^), rat (A^265^–L^295^), and Peking duck (S^253^–D^270^) ICL3.

The human CCK1R structure (PDB, 7F8X) [31] was used as a template to draw and compare with rat, mouse, and Peking duck CCK1R structure (Figure 6A). Other than transmembrane domains 1–7 (TM1–7), these four structures also share a similar short extracellular loop 1 (ECL1), bipartite β sheet-containing ECL2, horizontally oriented α helix-containing ECL3, short intracellular loop 1 (ICL1), twisted ICL2, a rather extensive ICL3, and the horizontal-oriented short α helix 8 at the C terminal (Figure 6A).

The human, rat, mouse, and Peking duck CCK1R structures overlap completely in the TM1–7, ECL1–3, and ICL1,2 (Figure 6B). Note, for example, complete overlap of the two β-sheets in ECL2, as well as the horizontal α helix in ECL3 (Figure 6B). Diversion or variation, however, could be noted especially in ICL3; progressively smaller loop (mouse > rat > Peking duck) in the secondary-structure-free or disordered portion of ICL3; the size of this region is similar in human and mouse, but seemingly with different orientations (bottom left part in Figure 6C).

**Figure 7:**
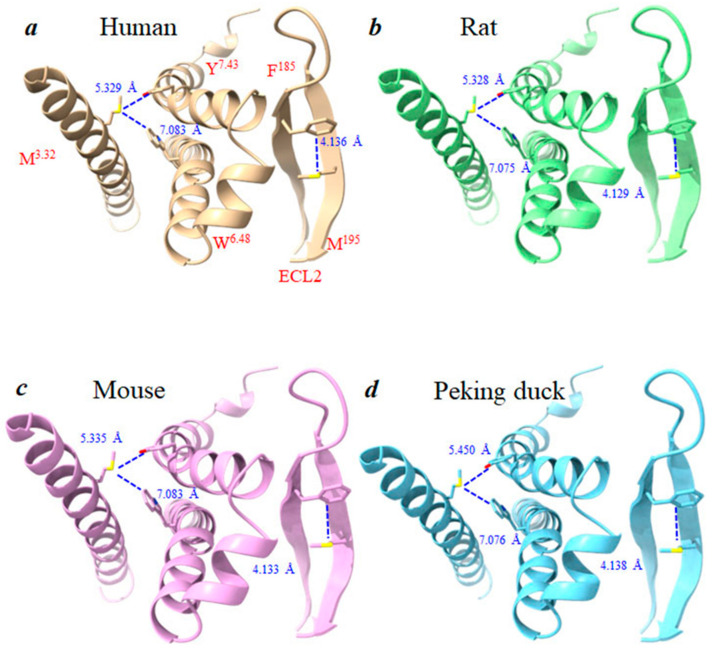
**The sulfur–aromatic interactions of M^3.32^ and Met^195/ECL2^ in human, rat, mouse, and Peking duck CCK1R.** (**a**) Human CCK1R 3D structure (PDB: 7F8X). Rat (**b**), mouse (**c**), and Peking duck (**d**) structures are made by Swiss-Model based on human CCK1R (PDB: 7F8X) as the template. Bond lengths of M^3.32^-W^6.48^, M^3.32^-Y^7.43^, and M^ECL2^-F^ECL2^ are each calculated with ChimeraX 1.3.

Of potential importance for photodynamic activation of human CCK1R, as noted before [14], ^1^O_2_-susceptible residues Met^121/3.32^ and Met^195/ECL2^ are shown in Figure 7. The sulfur–aromatic interactions (with Y^7.43^ + W^6.48^ and F^185^, respectively) could all be observed, but showed very little variation in bond length in human, rat, mouse, and Peking duck CCK1R: Met^3.32^-Y^7.43^, 5.329, 5.328, 5.335, 5.450; Met^3.32^-W^6.48^, 7.083, 7.075, 7.083, 7.076; M^193^-F^185^, 4.136, 4.129, 4.133, 4.138 Å (Figure 7).

## 4. Discussion

In the present work, photodynamic action with photosensitizer SALPC and red LED light was found to trigger persistent cytosolic calcium oscillations in the perifused rat, mouse, and Peking duck pancreatic acini, with a sensitivity order of mouse > rat > Peking duck. The photodynamically induced calcium oscillations were inhibited reversibly by CCK1R antagonist devazepide. Photodynamic action no longer induced any calcium increases after preincubation with ^1^O_2_ quencher Trolox C. Examination of predicted rat, mouse, and Peking duck CCK1R structures revealed a secondary-structure-free region in intracellular loop 3 (ICL3); the size of this region was correlated to the sensitivity to photodynamic activation of CCK1R: mouse > rat > Peking duck. The photodynamic activation motif YFM identified previously in human CCK1R was found to be conserved in rat, mouse, and Peking duck CCK1R.

### 4.1. Evolution of CCK-Stimulated Activation of CCK1R

Immunocytochemistry localized CCK1R to the basolateral plasma membrane in rat, mouse, and Peking duck pancreatic acini (Figure 1, Figure 2 and Figure S1). This subcellular localization is consistent with previous works carried out with rat and mouse pancreatic acini [37,38]. The anti-peptide antibody used in the present work was raised against human CCK1R^242−257^ (FEASQKKSAKERKPST) (https://www.abcam.com/cck1-r-antibody-ab77269.html, accessed 2 January 2023), which is within ICL3 (Figure 5). Although the human, rat, mouse, and Peking duck sequences are not identical in this small segment of 16 residues, the sequence homology is reasonably high (Figure 5). This intracellularly targeted antibody could tolerate variation in this region of ICL3 to recognize both mammalian and avian CCK1R (Figure 1, Figure 2 and Figure S1).

CCK stimulation of isolated rat, mouse, and Peking duck pancreatic acini (at 30, 10, and 300 pM, respectively) triggered regular calcium oscillations (Figure 1, Figure 2 and Figure S1). The CCK concentration needed in Peking duck pancreatic acini was much higher than in rat and mouse pancreatic acini (Figure 1, Figure 2 and Figure S1), with the sensitivity order of mouse > rat > Peking duck (Table 1). These data are consistent with previous reports [12,23,39]. Although Peking duck pancreatic acini were less sensitive to CCK stimulation, the receptor could be sensitized to be responsive to low picomolar CCK after treatment with cAMP-mobilizing secretagogues, such as the pituitary adenylate cyclase-activating peptide (PACAP) [23].

Although the complete preproCCK peptide sequence shows marked variation in human, rat, mouse, and mallard duck, the sulfated CCK octapeptide, which is used in most works, as in the present study, is completely identical (DYMGWMDF) (Figure 3). Therefore, this difference in CCK efficacy to activate CCK1R in rat, mouse, and Peking duck pancreatic acini must rest with the CCK1R receptor protein themselves.

To compare the protein sequence of human, rat, mouse, and Peking duck CCK1R, we cloned for the first time the *CCK1R* gene from Peking duck pancreatic acini. The CCK1R gene sequence of the mallard duck (*Anas platyrhynchos*) was used to design primers for RT-PCR amplification from Peking duck (*Anas platyrhynchos domestica*) mRNA (Figure 4). The cloned Peking duck CCK1R gene was uploaded to the NCBI database (MN250295.1) (Figure 4). The inferred Peking duck CCK1R protein sequence (QIJ58473.1) was found to be completely identical to mallard duck CCK1R, but the coding sequences were not identical; the last digit in the codon triplets showed variation for residues Asp^17^, Leu^46^, Val^128^, Ala^161^, Ser^262^, and Leu^384^.

The comparison of human, rat, mouse, and Peking duck CCK1R protein sequences (Figure 5, Table 2) revealed that, of residues important in CCK-recognition, binding, and receptor activation [27,28,29], most are identical. However, human W^39/1.30^, Q^40/1.31^, and L^356/7.39^; rat W^54/1.30^, Q^55/1.31^, and L^372/7.39^; and mouse W^39/1.30^, Q^40/1.31^, and L^364/7.39^ were found to be changed to L^42/1.30^, H^43/1.31^, and H^358/7.39^ in Peking duck CCK1R (Figure 5; Table 2). Because, in human CCK1R, W^39/1.30^, Q^40/1.31^, and L^356/7.39^ are CCK octapeptide-binding residues, and their mutations could decrease CCK-binding affinity 10-, 10-, and 8-fold, respectively [28]; this may be the reason why Peking duck CCK1R was less sensitive than either rat or mouse CCK1R (Figure 1, Figure 2 and Figure S1; Table 1).

Of CCK-binding-pocket-forming residues revealed by the solved complex structure of CCK-bound human CCK1R [31], human E^344/ECL3^, rat E^360/ECL3^, and mouse E^352/ECL3^ changed to Peking duck D^346/ECL3^, while human H^207/5.39^, rat Q^222/5.39^, and mouse Q^207/5.39^ changed to Peking duck Y^210/5.39^ (Figure 5; Table 2). This double mutation should also be correlated to decreased sensitivity of Peking duck CCK1R to CCK stimulation. However, the reason why mouse CCK1R is more sensitive than rat CCK1R remains to be explained (Figure 1, Figure 2 and Figure S1; Table 1).

The layered structural transitions (from extracellular to intracellular) of GPCR [32] were confirmed for human CCK1R [33]; the relevant residues involved are completely identical in human, rat, mouse, and Peking duck CCK1R (Figure 5, Table 2).

Of residues involved in Gq binding, most are identical in human, rat, mouse, and Peking duck CCK1R. The human I^296/ICL3^, rat I^312/ICL3^, and mouse I^304/ICL3^ residues were found to be changed to Peking duck residue A^298/ICL3^ (Figure 5, Table 2). As I^296/ICL3^ point mutation to G^296/ICL3^ was previously found to decrease human CCK1R binding to Gq [31], this could be the reason why Peking duck pancreatic acinar cell CCK1R should show decreased sensitivity to both CCK stimulated and photodynamic activation when compared with rat and mouse CCK1R (Figure 1, Figure 2 and Figure S1; Table 1).

### 4.2. Conserved Photodynamic Activation of CCK1R

Of the 20 amino acids made up of most proteins, only Met (M), Trp (W), Cys (C), His (H), and Tyr (Y) are susceptible to photodynamic ^1^O_2_ oxidation [35,36]. Out of the residues playing a key role in CCK-stimulated human CCK1R activation (Figure 5), only 10 are susceptible to ^1^O_2_ oxidation: C^94/2.57^, M^121/3.32^, Y^141/3.51^, Y^176/4.60^, M^195/ECL2^, C^196/ECL2^, H^207/5.39^, W^326/6.48^, Y^360/7.43^, and Y^370/7.53^. Of these 10 residues, 9 are completely identical in human, rat, mouse, and Peking duck CCK1R. This might be the reason why human [13], rat [10,11,12,19,20], mouse, and Peking duck CCK1R (this work; Figure 1, Figure 2 and Figure S1; Table 1) could all be photodynamically activated. The remaining critical residue (1 out of 10) in human CCK1R, H^207/5.39^, is changed to rat Q^222/5.39^, mouse Q^207.5.39^, and Peking duck Y^210/5.39^ (Figure 5, Table 2). This might partially account for the variation in sensitivity to photodynamic activation of rat, mouse, and Peking duck pancreatic acinar cell CCK1R, with the order of potency of mouse > rat > Peking duck (Figure 1, Figure 2 and Figure S1; Table 1). Note that both human H^207/5.39^ and Peking duck Y^210/5.39^ are ready targets for ^1^O_2_ oxidation, but Q^5.39^ (rat Q^222/5.39^ and mouse Q^207.5.39^) is often used in point mutation studies as an equivalent to oxidized Met [40,41]. The difference in Gq-binding I^296/ICL3^ (human I^296/ICL3^, rat I^312/ICL3^, and mouse I^304/ICL3^, but Peking duck A^298/ICL3^) (Figure 5, Table 2) might also account for this variation, but neither isoleucine nor alanine are susceptible to ^1^O_2_ oxidation [35,36].

Chemical photosensitizer SALPC was used in this work for photodynamic CCK1R activation in rat, mouse, and Peking duck pancreatic acini (Figure 1, Figure 2 and Figure S1). The mammalian and avian CCK1R receptors could also potentially be photodynamically activated with the genetically encoded protein photosensitizers, as confirmed with both human and rat CCK1R [13,14,15,19,20]. It may be noted here that SALPC is a widely used photosensitizer [24,42] with a high ^1^O_2_ quantum yield [43,44].

Both CCK-stimulated and photodynamic activation of rat, mouse, and Peking duck CCK1R were confirmed by reversible inhibition by CCK1R antagonist devazepide (Figure 1, Figure 2 and Figure S1). The potential involvement of ^1^O_2_ in photodynamic CCK1R activation in rat, mouse, and Peking duck pancreatic acini was corroborated by complete inhibition by ^1^O_2_ quencher Trolox C (Figure 1, Figure 2 and Figure S1), which has been widely used to confirm the involvement of ^1^O_2_ in photodynamic modulation [45,46,47].

The human, rat, mouse, and Peking duck CCK1R structures are highly similar in all regions, including TM1–7, ECL1–3, ICL1,2, and α helix 8; most diversity was seen in the extensive ICL3. These four CCK1R structures superimposed to a very high degree, especially in TM1–7, ECL1–3, and ICL1,2 (Figure 6). The highly similar structures likely underlie the conserved pharmacological property of photodynamic activation (Figure 1, Figure 2 and Figure S1; Table 1). The variation in ICL3 among rat, mouse, and Peking duck CCK1R (Figure 6), together with other differences (single point changes in critical residues; see above), could explain the ordered sensitivity to photodynamic activation (Table 1). Here, the progressively smaller loop in the disordered region of ICL3 was found (mouse > rat > Peking duck); the human loop was of similar size to the mouse, but with a different orientation (Figure 6C). To corroborate whether this decreasing order in size in the disordered region has any relevance in terms of liquid phase transition or phase condensation, and thus to Gq coupling efficiency and the order of sensitivity of CCK-stimulated and photodynamic CCK1R activation in rat, mouse, and Peking duck CCK1R, more experimentations will be needed in the future. Any solved rat, mouse, and Peking duck structures and their complex with Gq, under identical conditions to the human structure, will be most helpful.

### 4.3. Better Conservation of Photodynamic Activation of CCK1R

In photodynamic activation of pancreatic acinar cell CCK1R, although triggering rather similar cytosolic calcium oscillations as agonist CCK-stimulated CCK1R activation, greater variation in efficacy was seen in agonist-stimulated than photodynamic activation (Table 1) among rat, mouse, and Peking duck CCK1R (ratio ranging from 1 to 30 and from 1 to 1.6, respectively) (Table 1). Therefore, photodynamic activation is probably more conserved than agonist-stimulated CCK1R activation.

This difference between agonist-stimulated and photodynamic activation of CCK1R was also noted in previous studies with human CCK1R tagged with N-terminal protein photosensitizer miniSOG [15]. Of TM1–7 in human CCK1R, TM3 was especially important for receptor activation by both agonist stimulation and photodynamic activation. For Met^121/3.32^ in TM3 of human CCK1R, if mutated in miniSOG-tagged CCK1R (miniSOG-hCCK1R) to miniSOG-CCK1RM^121^A and miniSOG-CCK1RM^121^Q, EC5 values (a value of 5 in integrated calcium spike areas, per 10 min) increased from 21 pM to 150 and 760 pM respectively, with a decrease in CCK efficacy of 7 and 36 times, respectively [15]. In contrast, photodynamic activation with LED light irradiation (450 nm, 85 mW·cm^−2^, 1.5 min) decreased from 100% for wild type miniSOG-CCK1R, to 55% and 73% for miniSOG-CCK1RM^121^A and miniSOG-CCK1RM^121^Q, respectively, a decrease of less than one half (at 55%) or about a quarter (at 73%), respectively [15]. Therefore, for the human CCK1R, point mutations at Met^121/3.32^ affected agonist stimulation-induced receptor activation much more than photodynamic activation. This is rather similar to the difference between agonist-stimulated and photodynamic activation of CCK1R in rat, mouse, and Peking duck pancreatic acini.

Although agonist-stimulated CCK1R activation is likely more susceptible to receptor sequence variations than photodynamic CCK1R activation, both modes of receptor activation are likely to go through rather similar pathways of conformational changes, leading to Gq activation and elicitation of calcium oscillations. Protein photosenstizer miniSOG was tagged to human CCK1R at N-terminal, either directly, or via a linker of (GSG)*_n_* with different *n* numbers (*n* = 0, 4, 8, 12, ∞). The extended length of the linker between miniSOG and N-terminus of CCK1R in miniSOG-(GSG)_12_-CCK1R resulted in diminished miniSOG photodynamical CCK1R activation in comparison with miniSOG-CCK1R, miniSOG-(GSG)_4_-CCK1R, or miniSOG-(GSG)_8_-CCK1R [14], possibly because the longer linker of (GSG)_12_ affords more steric hindrance for both CCK-stimulated and miniSOG photodynamic activation of CCK1R, confirming that photodynamic human CCK1R activation may follow the same spatial conformational changes as CCK agonist activation of human CCK1R [14].

These previous works together with the present work suggest that photodynamic activation of CCK1R is better conserved than CCK-stimulated CCK1R activation. Then, it is interesting to note the photodynamic activation motif “YFM” we have identified previously [15].

### 4.4. Conserved Photodynamic Activation Motif “YFM” and Met-Aromatic Interactions

As mentioned above, TM3 is a critical pharmacophore for the photodynamic activation of human CCK1R, and the Y^119/3.30^F^120/3.31^Met^121/3.32^ motif is likely important for CCK1R activation by photodynamic ^1^O_2_ oxidation [15]. Examination of human, rat, mouse, and Peking duck CCK1R sequences confirmed the presence of the Y^3.30^F^3.31^M^3.32^ motif in all of them (Figure 5). Although the exact significance of this motif is not yet completely understood, Met residues have been noted for their regulation of both protein structure and function via sulfur–aromatic interactions [48,49]. Such interactions are present in human, rat, mouse, and Peking duck CCK1R; ^1^O_2_-susceptible Met^121/3.32^ interacts with aromatic Y^7.43^ + W^6.48^ and Met^195/ECL2^ with aromatic F^185^, respectively (Figure 7). Note the very similar bond length (Met^3.32^-Y^7.43^: 5.329, 5.328, 5.335, 5.450; Met^3.32^-W^6.48^: 7.083, 7.075, 7.083, 7.076; M^195^-F^185^, 4.136, 4.129, 4.133, 4.138 Å) in human, rat, mouse, and Peking duck CCK1R (Figure 7). These Met–aromatic interactions will impact photodynamic oxidative CCK1R activation and their conservation in mammalian and avian pancreatic acinar cell CCK1R.

## 5. Conclusions and Perspectives

**Figure 8:**
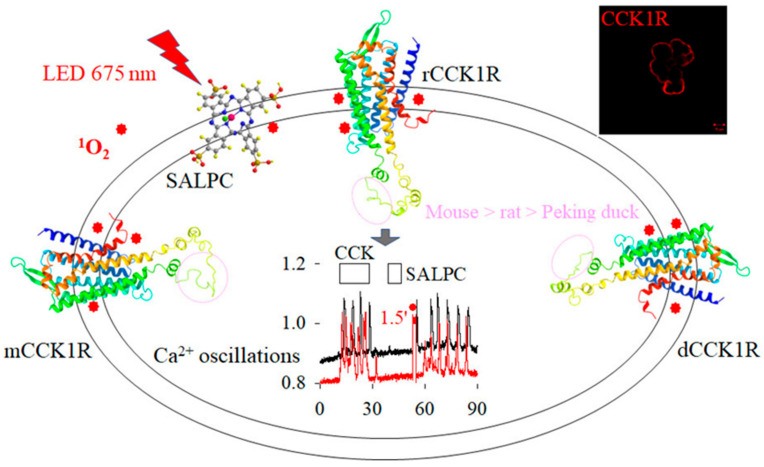
**Photodynamic CCK1R activation is conserved in mammalian and avian pancreatic acini.** CCK1R is present on basolateral plasma membrane in pancreatic acini (a single acinus is shown, top right, from Figure S1A), which is activated by both CCK stimulation and photodynamic action (SALPC, LED 675 nm, 1.5 min, from Figure S1Cb). Singlet oxygen (1O2) is likely the reactive intermediate in oxidative CCK1R activation. The structures of rat, mouse, and Peking duck CCK1R (rCCK1R, mCCK1R, and dCCK1R, respectively) are generated by Swiss-Model with the human CCK1R (PDB: 7F8X) as a template (from Figure 6A). The progressively smaller size of the structure-free portion (outlined by pink circles) in ICL3 (mouse > rat > Peking duck CCK1R) is correlated to the order of sensitivity for photodynamic CCK1R activation (mouse > rat > Peking duck). Abbreviations: CCK, cholecystokinin; LED, light-emitting diode; SALPC, sulphonated aluminum phthalocyanine. SALPC structure made with ChemDraw3D.

In conclusion, CCK1R is photodynamically activated in both mammalian and avian pancreatic acini, with the sensitivity order of mouse > rat > Peking duck. This sensitivity order is correlated to the decreasing size (mouse > rat > Peking duck) of the disordered portion of ICL3. Photodynamic CCK1R activation is better conserved in mammalian and avian species than CCK-stimulated CCK1R activation (Figure 8).

We have limited our present work to the isolated pancreatic acini. It is possible that CCK1R would show similar photodynamic activation in other expressing tissues, organs and cell types. *In situ* photodynamic CCK1R activation and their effect on organ/tissue function and, by extension, on overall organism neural and humoral signaling, would be interesting to study. Whether photodynamic CCK1R or CCK1-like receptor activation is limited to mammalian and avian species, or rather extended to other vertebrates or even to invertebrates, will need to be studied in the future. The clinical or medical implications of photodynamic CCK1R activation will be an important field of future studies.

After the submission of this work, a preprint has appeared that also stressed the importance of ICL3 in G protein coupling efficiency [50].

## Figures and Tables

**Table 1:** **Sensitivity of CCK-stimulated and photodynamic CCK1R activation in rat, mouse, and Peking duck pancreatic acini**.

Species	CCK Conc. Ratio		Photodynamic Intensity Ratio	
Rat	30 pM	3	SALPC 1 µM; Light 60 mW.cm^−2^	1.2
Mouse	10 pM	1	SALPC 1 µM; Light 50 mW.cm^−2^	1
Peking duck	300 pM	30	SALPC 2 µM; Light 40 mW.cm^−2^	1.6

CCK stimulation at 10 pM in mouse pancreatic acini was defined as 1. Photodynamic intensity for mouse pancreatic acini SALPC 1 μM × light 50 mW·cm^−2^ = 50 μM × mW·cm^−2^ was defined as 1.0, then rat dose was 1.2 and Peking duck was 1.6.

**Table 2:** Difference in critical residues in CCK1R activation.

Residues for	Human	Rat	Mouse	Peking Duck
CCK recognition	E^344/ECL3^	E^360/ECL3^	E^352/ECL3^	D^346/ECL3^
CCK binding	W^39/1.30^ Q^40/1.31^ H^207/5.39^ L^356/7.39^	W^39/1.30^ Q^40/1.31^ Q^222/5.39^ L^372/7.39^	W^39/1.30^ Q^40/1.31^ Q^207/5.39^ L^364/7.39^	L^39/1.30^ H^40/1.31^ Y^210/5.39^ H^358/7.39^
Receptor activation	Completely identical			
G protein coupling	I^296/ICL3^	I^312/ICL3^	I^304/ICL3^	A^298/ICL3^

Compiled from [27,28,29,31].

## Data Availability

All data are included in this paper. The cloned Peking duck pancreatic acinar cell CCK1R gene sequence has been uploaded to NCBI database (MN250295.1).

## Supplementary Materials

The following supporting information can be downloaded at: https://www.mdpi.com/article/10.3390/biomedicines11030885/s1, Figure S1 and associated text description: Photodynamic CCK1R activation in mouse pancreatic acini.
