# Supplementary material for: Photodynamic Activation of Cholecystokinin 1 Receptor Is Conserved in Mammalian and Avian Pancreatic Acini
Source: Biomedicines. 2023 Mar 13;11(3):885. doi: 10.3390/biomedicines11030885 (PMC10046250; doi:10.3390/biomedicines11030885)
Supplement: Supplementary file 1 [file biomedicines-11-00885-s001.zip › biomedicines-2180601-supplementary.pdf]

biomedicines

MDPI

Article

Photodynamic Activation of Cholecystokinin 1 Receptor Is Conserved in Mammalian and Avian Pancreatic Acini

Jie Wang and Zong Jie Cui *

Institute of Cell Biology, Beijing Normal University, Beijing 100875, China; 15321227265@126.com
* Correspondence: zjcui@bnu.edu.cn; Tel.: +86-10-5880-9162

Supplemental Materials

Immunocytochemistry confirmed CCK1R localization at the basolateral plasma membrane in isolated mouse pancreatic acini (Fig. S1*A*).

In perifused mouse pancreatic acini, CCK 1 pM was found to have no effect, but CCK 3 and 10 pM induced conspicuous calcium oscillations, CCK 30 pM produced a phasic increase (Fig. S1***Ba***). Continued perfusion with CCK 10 pM triggered regular calcium oscillations (Fig. S1***Bb***). A clear dose-response curve for CCK stimulation was found (Fig. S1***Bc***).

After CCK 10 pM-induced calcium spikes in mouse pancreatic acini were washed out, the addition of SALPC 1 μM alone had no effect on basal calcium (Fig. S1***Ca***). However, after sequential CCK 10 pM (calcium oscillations) and SALPC 1 μM (no effect), red LED light (675 nm, 50 mW·cm^{-2}, 1.5 min) elicited fresh calcium oscillations (Fig. S1***Cb***). If experiment shown in Fig. S1***Cb*** (sequential CCK 10 pM; SALPC 1 μM; 675 nm, 50 mW·cm^{-2}, 1.5 min) was repeated in the presence of $^1O_2$ quencher Trolox C 300 μM, no discernible calcium spikes were observed after LED light irradiation, although subsequent CCK 10 pM induced robust calcium oscillations (Fig. S1***Cc***). Statistical analysis of data from multiple experiments confirmed that SALPC photodynamic action (SALPC 1 μM, 675 nm, 50 mW·cm^{-2}, 1.5 min) triggered calcium increases, which were blocked by the presence of Trolox C (Fig. S1***Cd***, $P < 0.05$).

The calcium oscillations induced by photodynamic action (SALPC 1 μM; 675 nm, 50 mW·cm^{-2}, 1.5 min) were blocked reversibly by CCK1R antagonist devazepide 3 nM (Fig. 2***Ce***), with statistical significance ($P < 0.05$, Fig. S1***Cf***).

In control experiments, it was found that CCK 10 pM-induced calcium oscillations in mouse pancreatic acini were blocked by CCK1R antagonist devazepide 3 nM (Fig. S1***Cg***), but $^1O_2$ quencher Trolox C 300 μM was without any effect on CCK stimulation (Fig. S1***Ch***). Such difference was confirmed in statistical analysis (Fig. S1***Ci***).

SALPC therefore photodynamically activated mouse pancreatic acinar cell CCK1R, most likely via $^1O_2$.

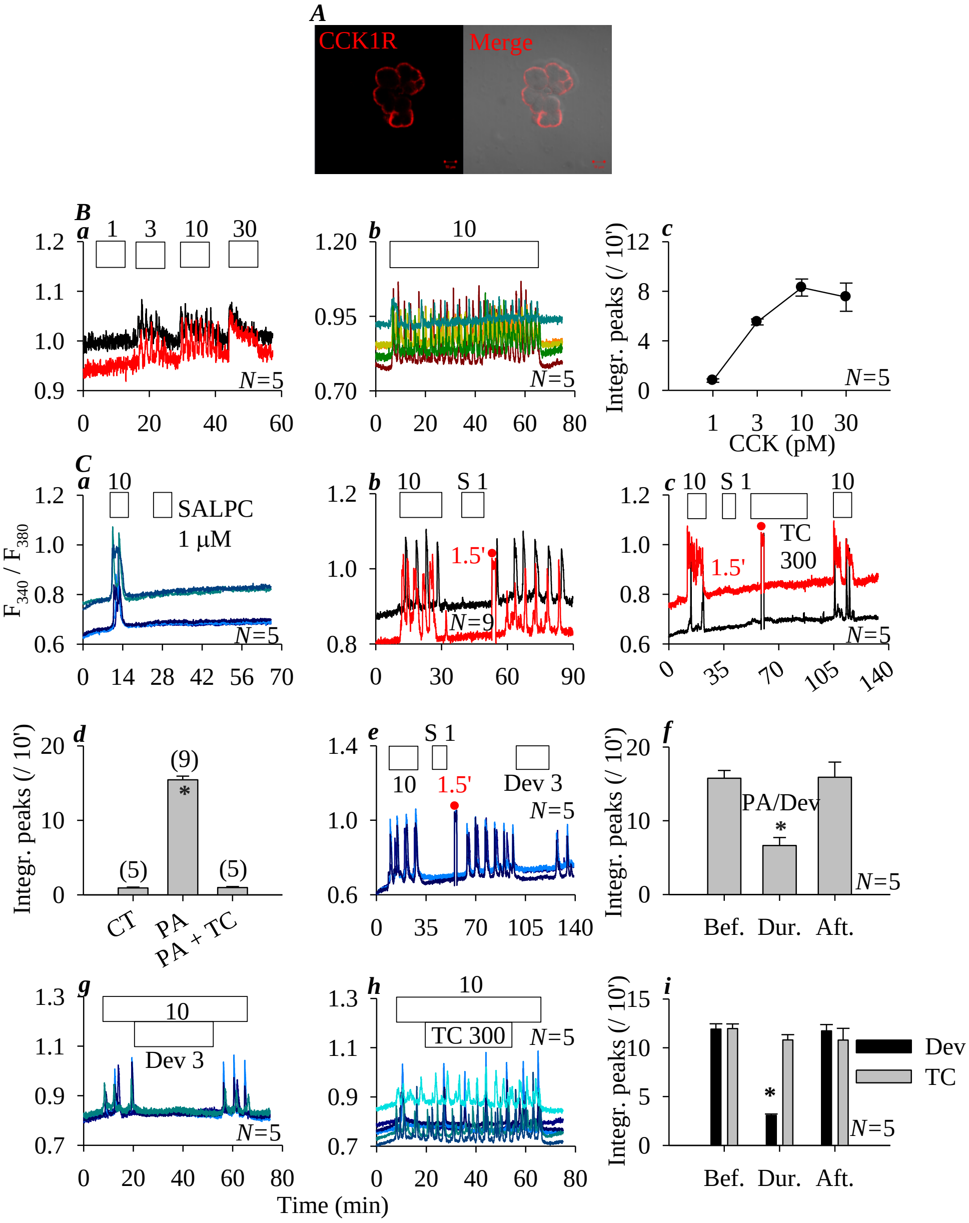

Figure S1. Photodynamic CCK1R activation in mouse pancreatic acini Pancreatic acini isolated from Kunming mouse were fixed and processed for immunocytochemistry, incubated with goat anti-human CCK1R[242-257] primary antibody, and subsequently with TRITC-conjugated donkey anti-goat secondary antibody. Shown are fluorescent (red) and merged (with bright field) images (***A***). Confocal images were taken in a Zeiss LSM 510 META (objective 63 × / 1.40 oil) with λ_{ex} 543 nm. Scale bars 10 µm. Pancreatic acini loaded with Fura-2 were perifused in Sykes-Moore chamber, CCK was added sequentially at 1, 3, 10, and 30 pM as indicated by the horizontal bars (***Ba***). Continued exposure to CCK 10 pM elicited regular calcium oscillations (***Bb***). The calcium peak areas above the baseline in typical original calcium tracings as shown in (***Ba***) were calculated (for 10 min) (5-15, 20-30, 35-45, and 50-60 min) from multiple repeat experiments and plotted in (***Bc***) (N = 5). CCK, SALPC, devazepide (Dev), Trolox C (TC), and LED light (675 nm, 50 mW·cm^{-2}, 1.5 min) were applied as indicated by the horizontal bars in (***Ca-Cc***). (***Ca***) CCK 10 pM, SALPC 1 µM. (***Cb***) CCK 10 pM, SALPC 1 µM, LED light (1.5 min). (***Cc***) CCK 10 pM, SALPC 1 µM, Trolox C (TC) 300 µM, LED light (1.5 min), CCK 10 pM. Integrated calcium peaks (above baseline) during (35-65 min in ***Ca***, 55-85 min in ***Cb***, 65-95 min in ***Cc***) were calculated from multiple repeat experiments (*N* = 5-9) and statistical data presented (***Cd***). (***Ce***) CCK 10 pM, SALPC 1 µM, LED light (1.5 min at red dot), devazepide (Dev) 3 nM. Integrated calcium peak areas above baseline (per 10 min) in calcium tracings as shown in (***Ce***) before, during, and after devazepide (Dev) were calculated from multiple repeat experiments (*N* = 5) and statistical data presented in (***Cf***). (***Cg***) CCK 10 pM, devazepide (Dev) 3 nM; (***Ch***) CCK 10 pM, Trolox C (TC) 300 µM. Integrated calcium peak areas above baseline (per 10 min) in calcium tracings shown in (***Cg***, ***Ch***), before, during, and after devazepide or Trolox C were calculated from repeat experiments (*N* = 5) done on different days with pancreatic acini isolated from different mice, and statistical data presented in (***Ci***). Student's T-test was performed, asterisk (*) indicates statistical significance at $P < 0.05$ (***Cd***, ***Cf***, ***Ci***, *N*= 5-9). The original calcium tracings are each from one of N identical experiments. Each color-coded trace is representative of one individual pancreatic cell (as one region of interest – ROI, in the optical field). The abscissa in panels (***Ba***, ***Bb***, ***Ca-Cc***, ***Ce***, ***Cg***, ***Ch***) are time in min as indicated.
